# Experimental Verification of Micro-Doppler Radar Measurements of Fall-Risk-Related Gait Differences for Community-Dwelling Elderly Adults

**DOI:** 10.3390/s22030930

**Published:** 2022-01-25

**Authors:** Kenshi Saho, Masahiro Fujimoto, Yoshiyuki Kobayashi, Michito Matsumoto

**Affiliations:** 1 Department of Intelligent Robotics, Toyama Prefectural University, Imizu 939-0398, Toyama, Japan; 2 Human Augmentation Research Center, National Institute of Advanced Industrial Science and Technology, Kashiwa 277-0882, Chiba, Japan; masahiro-fujimoto@aist.go.jp (M.F.); kobayashi-yoshiyuki@aist.go.jp (Y.K.); 3 Toyama College of Welfare Science, Imizu 939-0341, Toyama, Japan; matsumoto@urayama.ac.jp

**Keywords:** micro-Doppler radar, gait measurement, fall risk, faller classification, elderly people, support vector machine

## Abstract

In a previous study, we developed a classification model to detect fall risk for elderly adults with a history of falls (fallers) using micro-Doppler radar (MDR) gait measurements via simulation. The objective was to create daily monitoring systems that can identify elderly people with a high risk of falls. This study aimed to verify the effectiveness of our model by collecting actual MDR data from community-dwelling elderly people. First, MDR gait measurements were performed in a community setting, and the efficient gait parameters for the classification of fallers were extracted. Then, a support vector machine model that was trained and validated using the simulated MDR data was tested for the gait parameters extracted from the actual MDR data. A classification accuracy of 78.8% was achieved for the actual MDR data. The validity of the experimental results was confirmed based on a comparison with the results of our previous simulation study. Thus, the practicality of the faller classification model constructed using the simulated MDR data was verified for the actual MDR data.

## 1. Introduction

Falling is a common occurrence in elderly adults, and it is a leading cause of morbidity and disability. As most falls that result in injuries occur during walking [1], daily monitoring systems are required for elderly adults to prevent future falls. For this purpose, a method of assessing fall risk using gait must be developed. The gait information that shows the differences in fall risk can be useful for screening people with high fall risk. Numerous studies have verified that fall risk varies significantly with the gait of different types of people, such as the young, elderly, and those with a history of falling (elderly fallers) [2,3,4]. In particular, the fall risk of elderly fallers is considerably larger than that of healthy elderly people. Thus, the early detection of fall risk in elderly adults is essential for reducing and preventing critical accidents due to falling [4].

Most conventional studies have used optical motion capture techniques [2,5,6] or accelerometry [3,5,7,8,9] to perform detailed gait analysis, which measures the gait parameters related to fall risk. However, these techniques require participants to wear sensors or markers, and thus they are unsuitable for daily gait assessment. Although various wearable sensors with sufficient comfortability to the users have been proposed [10,11,12], these techniques also require the users to wear specific devices. Therefore, the remote sensing technique is promising to develop simpler systems that do not require wearing any devices. Optical sensing techniques, such as depth sensors and camera-based approaches, have been widely studied in recent years to remotely measure gait information [13,14,15,16,17]. The effectiveness of the depth sensors-based gait measurements has been verified for the differences in gait parameters for the subjects with stroke or elderly fallers [13,14]. In [15], the level of frailty was estimated for elderly people using a depth sensor to measure various motions including walking. Furthermore, several gait parameters, such as the gait speed and cadence, have been measured to a certain extent using a smartphone camera and depth sensor [16]. In [17], classification of fall risks using video cameras was performed. However, their accuracy depends on lighting conditions and the clothing of subjects. In addition, all the above methods have a major problem in that they cannot directly measure the velocity information of human body parts in gait.

Micro-Doppler radar (MDR) is a promising solution to these problems. MDR can remotely measure the velocity of entire human body parts without placing any constraints on participants [18,19,20]. There are no limitations of lighting conditions and clothing. Moreover, MDR devices are sufficiently small for daily monitoring in homes, hospitals, senior day care centers, rural community centers, etc. The effectiveness of MDR for gait classification has been verified in rehabilitation and hospital applications [21,22,23]. In addition, detailed gait analysis methods based on biomechanical approach using MDR have recently been developed [24,25]. In recent years, machine-learning-based approaches that use MDR data have been employed to recognize various types of motion [26,27]. In particular, MDR-based fall detection systems have been developed, and their effectiveness has been demonstrated in realistic situations [28,29,30]. However, these systems aim to detect fall events and do not detect fall risk. To prevent future falls, fall risk must be detected using gait information before a fall occurs.

In our previous study, we developed a method for detecting fall risk without the direct measurement of fall events. We utilized the MDR-based gait classification of healthy young and elderly adults based on the gait parameters related to their fall-risk-related gait differences [31,32,33], indicating the possibility of remotely measuring gait differences. In [31], we demonstrated the gait classification of young (aged in their 20s) and elderly (65 years and older) groups with over 90% accuracy using a recurrent neural network with time–velocity signals extracted from MDR data. However, the detection of elderly fallers is more important for fall prevention. We presented the classification of elderly fallers using only simulated MDR data [32,33]. To our best knowledge, our previous paper [32] is the first report on the radar-based faller classification (not the detection of fall events) and there are no other studies except for the authors’ recent work [33] that deal with this classification problem. We constructed a support vector machine (SVM) model using the kinematic gait parameters to classify elderly fallers and non-fallers [33]. We achieved a classification accuracy of 78%, which was better than that of a deep-learning-based model. Furthermore, the efficient gait parameters for faller classification were clarified (e.g., leg velocity in leg swinging motion during walking). In [33], simulated MDR data were generated using the AIST Gait Database 2019 [34], which is composed of the optical motion capture data of various participants including fallers. These data were used for model construction, validation, and the clarification of efficient gait parameters. However, the drawback of this method was that the simulation-based examination and the acquisition of the data in the AIST Gait Database 2019 were conducted in laboratory settings, and realistic situations (e.g., community settings) were not considered. Thus, it is necessary to validate our gait classification method for elderly fallers/non-fallers using MDR data collected in an actual environment.

This study demonstrates the gait classification of elderly non-fallers and fallers using actual MDR data collected from community-dwelling elderly participants. Based on our previous study [33], the classification model was developed using the simulated MDR data and was used to classify the actual MDR data. The classification model used the SVM whose feature parameters were the gait parameters extracted from received MDR signals. These parameters were determined to be efficient for detecting fallers. The contributions of this study are summarized below.

- The effectiveness of the classification model constructed based on simulated MDR data was verified using actual MDR data. Thus, this study experimentally verified our previous simulation study.
- The actual MDR data of elderly fallers were collected via experiments in a community setting (not in a laboratory setting). In other words, similar data on the practical use of monitoring systems for community-dwelling elderly adults were collected.
- A classification accuracy of 78.8% was achieved for the actual data through the training and validation process of the classification model using only the simulated data.
- The comparison of the results of gait parameter extraction and classification based on the simulated and actual data indicated the validity of both types of data.

The remainder of this paper is organized as follows: Section 2 describes the experimental participants, MDR experiments used to generate datasets, classification procedures, and analyses. Section 3 presents the classification results and clarifies the contribution of this study. Finally, Section 4 highlights the main contributions of this study and discusses topics for future research.

## 2. Methods

### 2.1. Participants and Experimental Protocol

Experimental MDR data were collected in a community setting, i.e., at senior day care centers and a health checkup event held at a rural community center. The participants for the MDR gait measurement experiments were 33 community-dwelling elderly adults aged 65 years and above. The participants responded to a questionnaire about the history of falls within a year. We used this questionnaire to classify the participants into the non-faller group (19 people; 2 men; mean age: 78.8 ± 7.06 years; mean height: 150.8 ± 9.66 cm) and faller group (14 people; 2 men; mean age: 82.5 ± 3.90 years; mean height: 150.6 ± 9.16 cm). There were no significant differences in the age and height between the two groups (Welch’s *t*-test results: *p* > 0.05).

All participants were able to walk without the assistance of another person or walking aids. The participants walked for 10 m, and their gait was measured using MDR. Then, we extracted the gait parameters that are known to be efficient for the fallers/non-fallers classification from the measured MDR data. The details of these experiments and parameter extraction methods are described in Section 2.2. Finally, the classification accuracy of the model for the collected experimental data was evaluated; this is explained in Section 2.3.

### 2.2. MDR Gait Measurement and Gait Parameter Extraction

**Figure 1:**
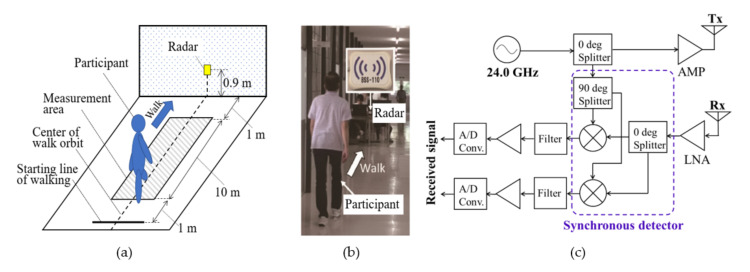
Micro-Doppler radar (MDR) sensing system for gait measurement. (**a**) Measurement system model; (**b**) Experimental site; (**c**) Block diagram of the MDR.

An MDR gait measurement system similar to that utilized in [31] was used in this study. Figure 1 shows the MDR system setup and experimental site. The participants walked toward the MDR system along a 10 m walkway (with 1 m areas for acceleration before walking and deceleration before stopping) at a self-determined comfortable pace. The walkway was flat, and no restrictions were imposed on the type of clothes and shoes. None of the participants wore unstable footwear such as high heels or slippers or used a walking aid.

The commercial continuous-wave MDR (ILT Office Inc., Toyama, Japan, BSS-110) operating at 24.0 GHz frequency was used. The specifications of the MDR system are summarized in Table 1, and the block diagram of the used radar system is shown in Figure 1c. The MDR system was composed of two antennas and transmitted a continuous sinusoidal wave with a frequency of *f*_0_ = 24.0 GHz to a walking participant. The signals received after synchronous detection *s*(*t*) were composed of the Doppler frequencies *f*_d_ corresponding to the Doppler velocities *v*_d_ = *cf*_d_/(2*f*_0_) (*c*: speed of light) of the scattering centers on the body parts, such as the legs and torso [18,19] (i.e., the Fourier transform of *s*(*t*) is expressed as S(*f*) = ∑*A*_i_*δ*(*f*−*f*_d_*_i_*) where *δ*(*f*) is Dirac’s delta function and *A*_i_ and *f*_d_*_i_* are received amplitude and Doppler frequency of *i*-th scattering center). We collected the data of the participants in the measurement area shown in Figure 1a. These data corresponded to steady-state walking. The sampling frequency of demodulated received signals was set as 600 Hz, which corresponded to the maximum measurement velocity of 3.75 m/s. This velocity was sufficient for measuring the walking motion, including the motions of toes during the leg-forward motion. The beamwidth and effective isotropic radiated power were set to measure the data for the entire body and measurement area.

**Figure 2:**
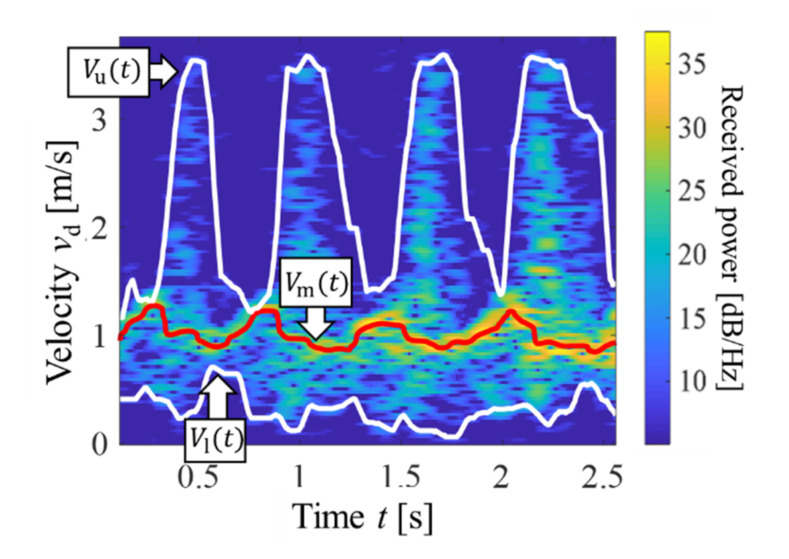
Representative spectrogram of experimental MDR data for gait measurement. *v*_d_ is the Doppler velocity calculated using the Doppler frequency.

The gait parameters were extracted by generating the spectrograms (time–velocity distributions) of the received MDR signals using the short-time Fourier transform (STFT) |*S*(*t*, *f*_d_)|^2^ = |∫*s*(*τ*)*w*(*τ*−*t*)exp(−j2π*f*_d_*τ*)d*τ*|^2^ (*w*(*t*): window function) [31]. Before the calculations of the STFT, we applied a high-pass filter to the received MDR signals to eliminate the components corresponding to a Doppler frequency of 0 Hz. These components corresponded to echoes from static targets. The high-pass filter was a one-dimensional Butterworth filter with a cutoff frequency of 20 Hz, which corresponded to a Doppler velocity of 0.125 m/s. Then, we applied the STFT with a Hamming window function with a width of 128 samples (213 ms) and an overlap length of 127 samples. Figure 2 shows an example of the spectrogram of the measured MDR data. The figure clearly shows the features of the Doppler velocities of the walking motion [24]. For example, the maximum velocity component for each time corresponds to body velocity in gait and includes information on walking speed [25]. We used the process employed in our previous study [33] to extract the upper envelope *v*_u_(*t*) (significant peaks corresponding to the maximum Doppler velocity for each time *t*), lower envelope *v*_l_(*t*) (significant peaks corresponding to minimum Doppler velocity for each *t*), and mean envelope of *v*_m_(*t*) (power-weighted mean velocity for each *t*), as shown in Figure 2. *V*_u_(*t*), *v*_l_(*t*), and *v*_m_(*t*) correspond to leg-forward motion, motion of legs in contact with the floor, and body motion, respectively (information of the arm motions is less, or not, included in these envelopes because the powers of the reflected echoes of the arms were relatively weak and the previous study confirmed that *v*_u_(*t*) almost corresponds to the movements of toes [20,25]). These envelopes were used to extract the following four gait parameters, which were determined to be efficient for the fallers/non-fallers classification in our previous simulation study [32,33]:

- The mean body velocity, *v*_m,mean_ = E[*v*_m_(*t*)], where E[ ] indicates the mean with respect to *t*.
- The mean leg velocity during the leg-forward motion, *v*_u,mean_ = E[*v*_u_(*t*)].
- The degree of variation in leg velocities during the leg-forward motion, *v*_u,std_ = STD[*v*_u_(*t*)], where STD[ ] indicates the standard deviation with respect to *t*.
- The degree of variation in leg velocities in the stance phase, *v*_l,std_ = STD[*v*_l_(*t*)].

### 2.3. Gait Classification Based on SVM Model Constructed Using Simulated MDR Dataset

To classify the fallers/non-fallers using the MDR data, a gait-parameter-based classification model was constructed using the SVM and simulated MDR dataset used in our previous study [33]. Then, the constructed classification model was evaluated for the gait parameters extracted from the actual MDR data. In other words, the simulated data were used for training and validation and the actual data were used for testing.

The classification model was constructed using the gait parameters (*v*_m,mean_, *v*_u,mean_, *v*_u,std_, *v*_l,std_). Note that our conventional study used the twelve gait parameters presented in [33]. However, this study used only four parameters because they were determined to be efficient for the faller classification problem, and a simpler model was preferred for practical use. The Gaussian kernel function was selected for the SVM because it is a general-purpose function [35]. The classification hyperplane parameters were determined by utilizing a soft-margin optimization process, and the SVM parameters were optimized via a validation process by performing a grid search [36]. We investigated the classification performance for the two SVM models: the model whose parameters were optimized for the accuracy and the model whose parameters were optimized for the sensitivity. We used 480 simulated MDR data points generated from the AIST Gait Database 2019 for training and validation. A total of 70% of the data were used for training, and the remaining 30% for validation.

Finally, classification of the actual MDR data was performed using the constructed SVM model, and its accuracy was evaluated. Thirty-three experimental data of the participants described in Section 2.1 were used as the test data.

## 3. Results and Discussion

### 3.1. Gait Parameter Extraction

**Figure 3:**
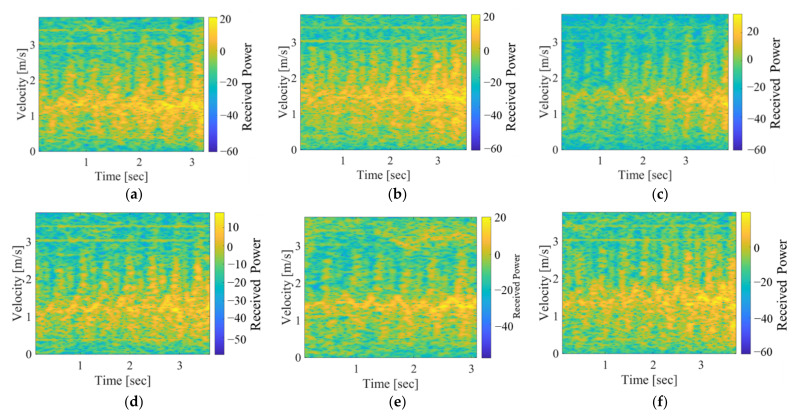
Examples of the measured spectrograms of six participants. (**a**–**c**): Non-fallers; (**d**–**f**): Fallers.

**Figure 4:**
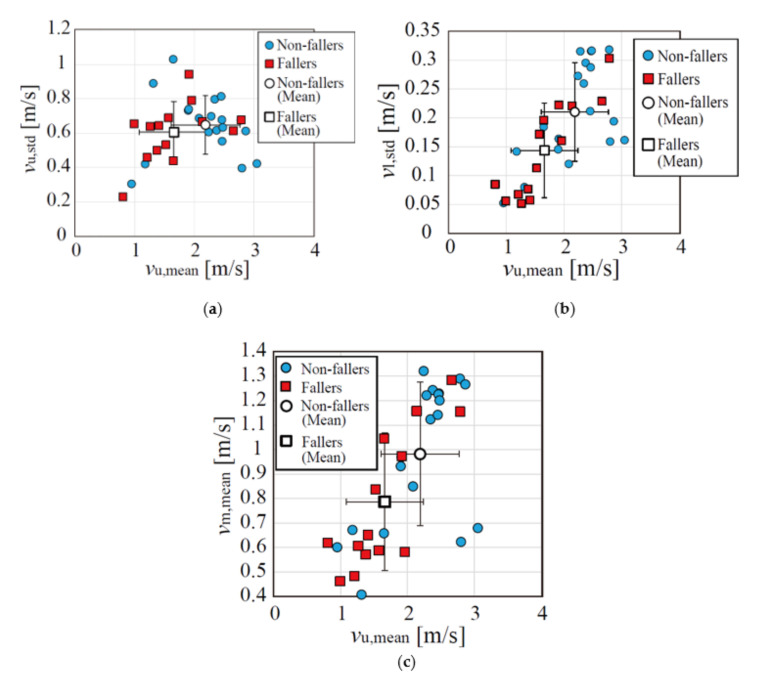
Gait parameters extracted from the experimental data. (**a**) *v*_u,std_ vs. *v*_u,mean_; (**b**) *v*_l,std_ vs. *v*_u,mean_; (**c**) *v*_m,mean_ vs. *v*_u,mean_.

Figure 3 shows the examples of the extracted spectrograms of six participants. We can see the slight tendency that the velocities of the fallers were smaller than those of the non-fallers. However, the differences are unclear. Thus, we extracted the gait parameters to classify the data of both groups. Table 2 summarizes the four gait parameters extracted from the experimental MDR data, and Figure 4 shows the plots of all parameters. All parameters of the non-faller group were larger than those of the faller group. Significant differences were observed only for *v*_u,mean_ and *v*_l,std_ (significance level was 0.05). However, as shown in Figure 3, the divergence of the two groups was confirmed to a certain extent, particularly in the plots of *v*_u,mean_ and *v*_u,std_. Thus, non-fallers and fallers were classified using these parameters.

**Figure 5:**
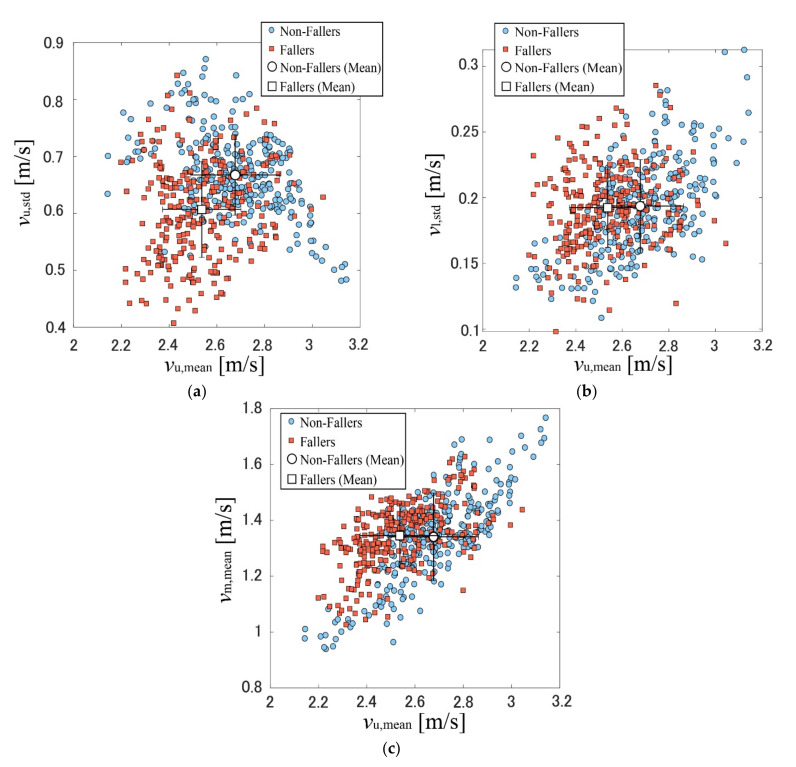
Gait parameters extracted from the simulated data [33]. (**a**) *v*_u,std_ vs. *v*_u,mean_; (**b**) *v*_l,std_ vs. *v*_u,mean_; (**c**) *v*_m,mean_ vs. *v*_u,mean_.

Figure 5 shows the plots of the gait parameters obtained from the simulated MDR data in our previous study [33]. The comparison of Figure 4 and Figure 5 shows similar trends for the parameters obtained from the experimental and simulated data. However, the parameters obtained from the experimental data were relatively smaller than those obtained from the simulated data. This was because the data used for the simulations, including the AIST Gait Database 2019, were collected in laboratory settings [34]. In contrast, the experimental data were collected in an actual community setting. These results can be considered valid because the gait pattern is affected by the surrounding environment [37]. Furthermore, the age and gender in the simulated and experimental data were different: the number of male participants was quite small, and the ages of the participants in this study were larger than those in our previous simulation study with mean age of approximately 67 years. However, as the parameters obtained using the experimental and simulated data showed similar trends, the classification model constructed using the simulated data can be applied to the experimental data.

### 3.2. Classification Results

First, we investigated the classification performance using the model tuned for the classification accuracy. Table 3 shows the results of the classification test conducted using the actual MDR data. The classification accuracy, sensitivity, specificity, and precision were 78.8%, 64.3%, 81.8%, and 89.5%, respectively. Although the sensitivity was not relatively high, the other classification indices were sufficiently better. The classification accuracy was similar to our simulation results (the classification accuracy for the simulated data was 78.9% [33]). Then, we investigated the performance using the model tuned for the sensitivity, and Table 4 shows the results. The classification accuracy, sensitivity, specificity, and precision were 72.7%, 71.4%, 66.7%, and 73.7%, respectively. Over 70% sensitivity was achieved for this model. However, other performance indices were relatively worse. Thus, when the sensitivity is important for the assumed application, this model is efficient. These results demonstrated that the classification model constructed using the simulated MDR data was effective for the actual MDR data. Furthermore, the effectiveness of the gait parameters, which may reflect the fall risk during walking, was experimentally demonstrated.

### 3.3. Comparison with Other Studies

This subsection discusses the differences between our study and the conventional studies on the faller classification including using other sensors and those on the radar-based gait measurements. Table 5 summarizes the conventional studies on the faller classification using various sensors. For the accelerometry technique, a relatively large number of studies have been reported. However, as described in Section 1, these require the contact of the sensor devices to the human body. Thus, the use of remote sensors has the advantage of practicality. However, the number of studies on the faller classification using remote sensors, such as cameras, depth sensors, and radars, is quite small (note that although there are many reports on fall event detection, the classification of fall risks is not widely studied). Some studies, including [13], used depth sensors and reported significant differences in the gait parameters between the fallers and non-fallers. However, their classification was not conducted. In [17], the video camera measured the Timed Up and Go Test, which is composed of standing up, walking, and sitting down movements, and fall risks of the participants were classified. However, its accuracy was 62.5%. In contrast, we achieved over 70% accuracy using the MDR remote sensing, which clearly indicates the effectiveness of our present study. Furthermore, as indicated in Table 5, our achieved accuracy and sensitivity values were acceptable compared with the accelerometry-based methods.

Next, we compared with other studies on the radar-based gait analysis [24,25]. These conventional studies proposed methods for efficient gait parameter extraction from MDR data. A similar extraction method was used in this study. However, the following three points are novel compared with these conventional studies on radar-based gait measurements:

- The conventional radar-based techniques including these conventional studies did not deal with the faller classification problem. Thus, the novelty of our previous and present studies is in performing the faller classification using the radar gait measurement.
- In [24,25], general gait parameters were extracted. In contrast, our study extracted the gait parameters related to the fall risks clarified in our study [32,33] and verified the effectiveness of the extracted parameters for the faller classification.
- The radar data of the actual community-dwelling elderly adults collected in the real environments (not in the laboratory settings) were used to show the feasibility of radar-based monitoring for daily healthcare applications.

### 3.4. Limitation of the Study

This study had three main limitations. First, the experiments were conducted for particular situations, and the number of male participants was quite limited. Furthermore, the ages of the participants were relatively large. For these reasons, the distributions of the simulated and experimental data were different (although their trends were matched to some extent) because the simulated data sufficiently included the various types of data. Thus, to improve the reliability of our study, further experiments should be conducted for a larger number of participants, including men and relatively younger participants, and various situations including homes and hospitals.

Second, because we used only the MDR, the data corresponding to the motion of arms were not sufficiently obtained (as explained in Section 2.2). The arm motions in gait also have the information of the gait changes, and acquisition of their parameters may improve the classification accuracy. To obtain arm motion information, other sensors can be used, such as inertial sensors. Furthermore, acceleration information, which is easily obtained using inertial sensors, is not directly obtained using the MDR. Thus, the sensor fusion of the inertial (acceleration) and MDR sensors can improve the classification accuracy and is an important research problem for the future.

Third, we assumed only the participants walking toward the MDR and those walking in other directions such as lateral directions were not considered. In the MDR measurements, the velocities along the radial direction only were obtained. Thus, it is difficult to accurately measure the participants walking in other directions using the single MDR. Thus, the experiments using multiple MDRs to measure the participants walking in arbitrary directions [20] is required for future study.

## 4. Conclusions

We performed MDR experiments on community-dwelling elderly participants for the early detection of fall risk in elderly adults using a remote monitoring system. Our aim was to verify the effectiveness of the model for the fallers/non-fallers classification constructed based on our previous simulation study [33]. Actual MDR data were collected from elderly adults in a community setting. The four gait parameters that were proven to be efficient for classification were extracted from the collected data. The SVM model for the classification was constructed using the gait parameters extracted from simulated MDR data. Thus, training and validation were performed using the simulated data. Then, testing was conducted using the actual data. The gait parameters extracted from the simulated and actual MDR data showed similar trends. We achieved a classification accuracy of 78.8% for the test process. These results verified the validity of our previous simulation study and effectiveness of the SVM model for the fallers/non-fallers classification in an actual environment.

Future experiments are needed to resolve the limitations described in the previous section. In addition, the classification accuracy and sensitivity can be improved by employing a data assimilation approach using simulated and actual data.

## Figures and Tables

**Table 1:** MDR specification.

Transmitting frequency	24 GHz
Transmitting waveform	Sinusoidal
Detector for received signals	Synchronous detector
Sampling frequency of received signals	600 Hz
Effective isotropic radiated power	40 mW
3 dB beamwidth	±35° (H-plane), ±14° (E-plane)
Physical size	6 cm (W), 2 cm (D), 7 cm (H)

**Table 2:** Extracted gait parameters.

Parameter	Fallers (Mean ± SD)	Non-Fallers (Mean ± SD)	*p* from Welch’s *t*-Test
*v*_m,mean_ (m/s)	0.787 ± 0.281	0.982 ± 0.293	0.0630
*v*_u,mean_ (m/s)	1.66 ± 0.578	2.19 ± 0.581	0.0151
*v*_u,std_ (m/s)	0.605 ± 0.170	0.647 ± 0.178	0.493
*v*_l,std_ (m/s)	0.144 ± 0.0817	0.210 ± 0.0854	0.0308

**Table 3:** Confusion matrix of the classification results of the test performed using actual data using the model tuned for the accuracy.

True\Predicted	Fallers	Non-Fallers
Fallers	9	5
Non-fallers	2	17

**Table 4:** Confusion matrix of the classification results of the test performed using actual data using the model tuned for the sensitivity.

True\Predicted	Fallers	Non-Fallers
Fallers	10	4
Non-fallers	5	14

**Table 5:** Comparison of conventional and present studies on the faller classification.

Study	Sensor (Other Conditions)	Sensor Type	Accuracy	Sensitivity
Daines et al. (2021) [7]	Accelerometer (smartphone, 6 min walk test)	Contact	81.3%	57.2%
Meyer et al. (2020) [8]	Accelerometer and gyro sensor (deep learning model, 1 min walk data)	Contact	86%	88%
Bet et al. (2021) [9]	Accelerometer ((measuring the timed up and go test)	Contact	75%	71%
Latorre et al. (2019) [13]	Depth sensor (Microsoft Kinect, 10 m walk test)	Remote	N.A. (Results of paired *t*-tests were only presented)	
Gandomkar et al. (2014) [17]	Video camera (measuring the timed up and go test)	Remote	62.5%	N.A.
This study	Doppler radar (model was tuned for the accuracy, 10 m walk test)	Remote	78.9%	63.2%
	Doppler radar (model was tuned for the sensitivity, 10 m walk test)		72.7%	71.4%

## Data Availability

The datasets of this study are not publicly available (no ethical committee approval) but might be available from the corresponding author on reasonable request and the authorization of other coauthors.
